# Impact of Social Determinants of Health on the Emerging COVID-19 Pandemic in the United States

**DOI:** 10.3389/fpubh.2020.00406

**Published:** 2020-07-21

**Authors:** Sravani Singu, Arpan Acharya, Kishore Challagundla, Siddappa N. Byrareddy

**Affiliations:** ^1^Department of Pharmacology and Experimental Neuroscience, University of Nebraska Medical Centre, Omaha, NE, United States; ^2^Department of Biochemistry and Molecular Biology, University of Nebraska Medical Centre, Omaha, NE, United States; ^3^Department of Genetics, Cell Biology, and Anatomy, University of Nebraska Medical Centre, Omaha, NE, United States

**Keywords:** SDOH, SARS-CoV-2, COVID-19, social inequality, public health, food, economy, education

## Abstract

A novel coronavirus (2019-nCoV) caused a global pandemic in the months following the first four cases reported in Wuhan, China, on December 29, 2019. The elderly, immunocompromised, and those with preexisting conditions—such as asthma, cardiovascular disease (CVD), hypertension, chronic kidney disease (CKD), or obesity—experience higher risk of becoming severely ill if infected with the virus. Systemic social inequality and discrepancies in socioeconomic status (SES) contribute to higher incidence of asthma, CVD, hypertension, CKD, and obesity in segments of the general population. Such preexisting conditions bring heightened risk of complications for individuals who contract the coronavirus disease (COVID-19) from the virus (2019-nCoV)—also known as “severe acute respiratory syndrome coronavirus 2” (SARS-CoV-2). In order to help vulnerable groups during times of a health emergency, focus must be placed at the root of the problem. Studying the social determinants of health (SDOH), and how they impact disadvantaged populations during times of crisis, will help governments to better manage health emergencies so that every individual has equal opportunity to staying healthy. This review summarizes the impact of social determinants of health (SDOH) during the COVID-19 pandemic.

## Introduction

The novel coronavirus (2019-nCoV) spread rapidly throughout China during the Chinese New Year in late January of 2020, a time of increased domestic and international travel for Chinese people. The first four cases of the novel coronavirus were reported on December 29, 2019. All four cases were linked to the Huanan Seafood Wholesale Market in Wuhan, a city with more than 11 million people and the capital of Hubei province in central China. The symptoms were described as a pneumonia of unknown etiology (1). Early cases show history of contact with the seafood market. Later and more recent cases were found to be transmitted via human-to-human contact (2). The disease caused by 2019-nCoV was named COVID-19 by the World Health Organization (WHO) on February 11, 2020 (3). The CDC confirmed that individuals with preexisting diagnoses of asthma, cardiovascular (CVD), hypertension, chronic kidney disease (CKD) and/or are elderly, immunocompromised, or obese have higher risk of severe illness from COVID-19 (4). Of the listed at-risk health demographics, asthma, CVD, hypertension, CKD, and obesity can be caused by discrepancies in socioeconomic status (SES). The CDC reports that 94% of patients who have died from COVID-19 had at least one preexisting condition (5). Because these conditions specifically put an individual at higher risk of being infected with SARS-CoV-2, these vulnerable populations must be given the resources needed to endure infectious outbreaks. This review summarizes the impact of social determinants of health (SDOH) during a pandemic of COVID-19. It can provide essential information to support the government's decision-making body to strategically manage health emergencies at community, national, and even international levels in the future if a similar situation was to arise. Calculated measures can be taken to prevent or reduce further transmissions in a vulnerable population that is at risk.

## Social Determinants of Health

**Figure 1:**
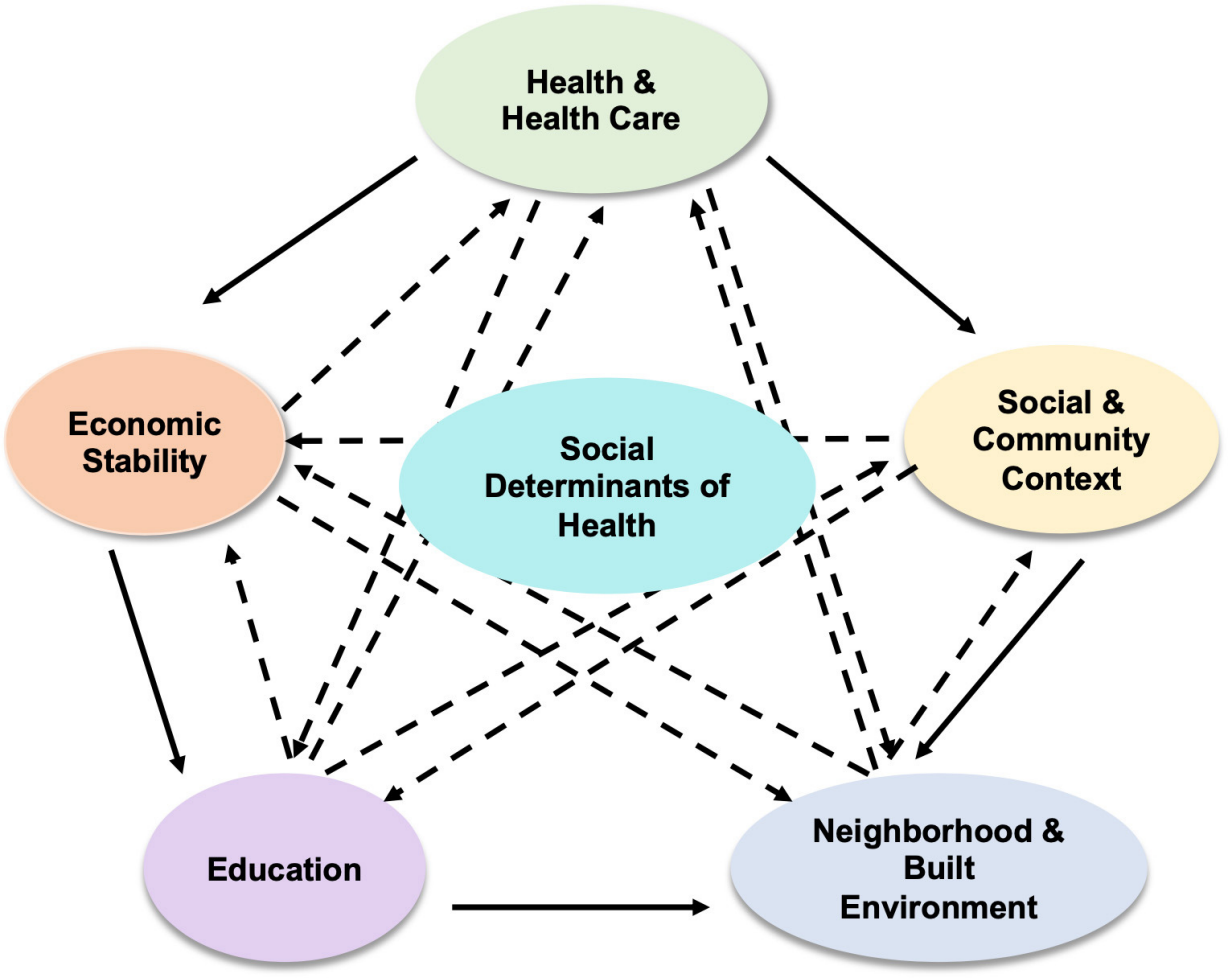
The five domains of social determinants of health (SDOH).

The social determinants of health (SDOH) are social and economic conditions that are categorized into five key determinants as summarized in Figure 1. Health and health care, social and community context, neighborhood and built environment, education, and economic stability (6). Health and health care include access to health care, access to primary care, health insurance coverage, and health literacy (7). Low health literacy can cause patients difficulty with navigating the complex healthcare system and understanding medical advice or prescriptions. Individuals without health insurance are less likely to utilize or even have access to primary care, which makes detecting and managing chronic conditions, such as CVD, asthma, diabetes, and cancer, difficult. Social and community context are the circumstances a person lives, learns, and works in. This domain of SDOH includes community involvement and discrimination. Lower mortality rates are associated with social and community support and cohesion. Neighborhood and built environment include housing, neighborhood, transportation, access to healthy foods, air quality, water quality, and access to green space (7). Air pollution has been shown to be associated with incident asthma. The CDC has confirmed that individuals with asthma are at higher risk for severe illness from COVID-19 (8). Safety plays a major role in health. People are more likely to walk or run outside if they feel safe in their neighborhood. Without the worry about crime and danger, safe neighborhoods also allow people to maintain good mental health. Immune function is influenced by psychological stress. Algren et al. state that individuals living in deprived neighborhoods were observed to have more stress when compared to those living in non-deprived neighborhoods. Stressors of those living in deprived neighborhoods include, “overcrowding, high crime rates, perceived danger, poor transportation, poor housing, disrepair, limited services, poor infrastructure, and a lack of social support” (9). Education includes high school graduation, enrollment in higher education, and language and literacy. The higher one's level of education, the higher his or her life expectancy is (7). It is important to disclose information regarding health in a patient-specific manner, taking into account the patient's education level. Economic stability includes employment, poverty, food security, and housing stability. The American Medical Association (AMA) states that as the poverty level increases, the percentage of adults who are 25 years and older with an activity-limiting chronic disease increases (7). Unemployment impacts an individual's health in many ways, as it has associations with depression, domestic violence, substance abuse, and physical illness.

Specific examples of SDOH include income, education, employment, and social support (10). Simply put, they are conditions into which one is born, grows, lives, works, and ages (11). They look at the person as a whole. Altogether, these conditions impact health status of individuals and communities. Disparities in any of these conditions are translated into a measure of social hierarchy called socioeconomic status (SES). The lower individuals are on the spectrum of SES, the poorer health outcomes they face. Due to poor outcomes, life expectancy decreases for those at the lower end of the spectrum (10). Socioeconomic inequality piles health complications on top of the financial woes already burdening disadvantaged segments of the population.

The five SDOH are interrelated and played major role during COVID-19 pandemic. For example, education level of an individual can impact his or her occupation, which determines economic stability and income level, which can impact the type of healthcare the individual is eligible for and what neighborhood the individual lives in, which then impacts the social and community context the individual is surrounded by and those factors played important role in current COVID-19 pandemic. Therefore, one can conclude that socioeconomic factors play a key role in infection and mortality rates. Specific examples include some county's in New York, such as Bronx, Brooklyn, and Queens have suffered higher mortality rate compared to other county's suggested that large of population of individuals with low economic status lived in these areas. Another example to consider is from the perspective of a child growing up in a family that does not have much economic stability. The child's parents have low-income jobs, which forces them to live in poverty-stricken neighborhoods that may not have a great school system. This child will not obtain the same quality of education as a child that lives in an affluent neighborhood that has a richer school district. Since, public schools in the U.S. are funded by local, state, and federal governments (12). Funding comes from income and property taxes. Affluent neighborhoods and districts collect more taxes; therefore, they have more funding. Low-income districts collect less funding and have substandard school facilities and teachers who are the least qualified (12). Therefore, below average quality of education will not lead to high college admission test scores, which will keep the child out of top colleges if he or she chooses to pursue a college education. Even with a low-tier college education, the child may not have many high-income job opportunities. This will land the child in the same position as his or her parents, with a low-income job living in a poverty-stricken neighborhood. Ham et al. (13) state that children living with their parents in poverty-stricken neighborhoods are more likely to end up in the same situation themselves later in their life. The five determinants can be thought of as a cycle of events that impact one another rather than as individual entities even in current COVID-19 pandemic.

## Health and Healthcare

### Health Literacy

Health literacy is defined by the U.S. Department of Health and Human Services (HHS) as “the degree to which individuals have the capacity to obtain, process, and understand basic health information needed to make appropriate health decisions” (14). This includes the ability to read and understand health-related pamphlets, prescriptions, written instructions from a healthcare provider, etc. Not being able to read or understand health-related information makes it difficult for individuals to take care of themselves, even if the awareness to do so is present. Low health literacy is associated with poorer health outcomes. Certain population groups have been noted to have low health literacy compared to other groups (14). Those who are living in poverty, not highly educated, from a certain race/ethnic group, or with disabilities are more likely to have low health literacy (14). Patients who demonstrate low health literacy may have high overall literacy and high verbal fluency, which causes the patient to present as having high health literacy. It is important to recognize people who may have low health literacy especially during times of a pandemic, because health literacy is an important means of preventing communicable diseases, such as COVID-19. Understanding infectious diseases to a certain degree, including mode of transmission and viability of pathogens, will help people readily accept the circumstances in situations like this rather than question the recommendations. Health literacy can allow people to understand their responsibility of adhering to social distancing and other recommended measures during the COVID-19 pandemic and the reasoning behind the measures being taken to prevent the spread of the virus.

A Gallup poll conducted in the months of April and May of 2020 looked at how many Americans considered social distancing to be significant by assessing their confidence level in the impact social distancing has on reducing the spread of COVID-19. Further, determined whether each group that was divided by confidence level followed social distancing. The study found that 54% of Americans were “very confident” and 31% were “moderately confident” in their belief that social distancing helps save lives during COVID-19 pandemic (15). However, 14% of Americans who participated expressed skepticism about social distancing and its role in saving lives. Overall, 88% of Americans who participated in the poll reported that they “always” or “very often” practiced social distancing, which included measures such as avoiding crowded places and leaving their homes unnecessarily. Of those who were “very confident” or “moderately confident” that social distancing makes a difference, 95 and 87% reported that they “very often” practiced social distancing, respectively. Fifty-seven percentage of those who expressed skepticism “very often” practiced social distancing. The percentages were drop when it comes to “always” practicing social distancing. Seventy-one percentage of those who were “very confident” that social distancing making a difference “always” practiced it, whereas 47% of those who were “moderately confident” “always” practiced it. Only 27% of those who were skeptical “always” practiced social distancing. Therefore, health literacy was played a major role in whether an individual understands a health emergency situation, such as COVID-19 pandemic, and whether he or she will follow recommendations, such as social distancing.

### Access to Health Care and Primary Care

Access to health care is described as the “timely use of personal health services to achieve the best possible health outcomes” by the National Academies of Sciences, Engineering, and Medicine (14). Many people face barriers to health care, which may hinder their ability to take responsible actions toward their well-being. Barriers include limited or no access to transportation for health appointments, lack of health insurance, limited education about health care, limited health care resources, provider hours limited to work hours, etc. Lack of health insurance is usually seen in populations with lower incomes and minorities. A study by Gallup and West Health found that 14% of adults in the U.S. revealed that they would not seek healthcare if they experienced a fever and dry cough (16, 17). Fever and dry cough are the most common symptoms of COVID-19. When adults were specifically asked whether they would seek healthcare if they had believed they had been infected with COVID-19, 9% still answered that they would not (16). The individuals that reported that they would not seek healthcare were non-white adults under the age of 30 who had a high school education or less earning less than a $40,000 income per year (16).

Reluctance to seek healthcare is associated with socioeconomic status. Hispanics and African Americans were less likely to have health insurance compared to non-Hispanic whites (16). Without health insurance, primary care visits may not be feasible, or people may hesitate to use health care resources. This puts those without health insurance at risk of not being screened for chronic conditions, such as CVD, hypertension, asthma, and diabetes. Access to health care also relies on the availability of resources (14).

Those who are minorities and/or have low incomes already face difficulty-accessing healthcare. Many of them primarily depend on student-run clinics for obtaining healthcare. The University of Nebraska Medical Center College of Medicine has a student-run clinic, called the Student Health Alliance Reaching Indigent Needy Groups (SHARING) clinic, which provides low-cost primary health care and services to the underprivileged populations in the Omaha community. This clinic has been closed due to the COVID-19 pandemic. Therefore, the underserved populations who already face barriers to healthcare now face a barrier to access primary care at these student-run clinics, which are their primary means of maintaining their well-being.

### Role of Food Deserts on Cardiovascular Disease

Food deserts are neighborhoods that are defined as low income areas with little access to healthy foods by the U.S. Department of Agriculture (USDA) (18). A study found that there was association between food deserts and cardiovascular risk factors in an Atlanta metropolitan area. They found that income was more strongly associated with CVD risk than access to healthy food (18). Recognizing that income had a greater part than location of residence, they then studied individual income vs. neighborhood income by observing people with low individual income living in low income neighborhoods and compared them with people with low individual income living in high income neighborhoods. Results showed that individual income is associated with higher risk of CVD than neighborhood income or food access. Those with high individual incomes who lived in low-income neighborhoods had lower CVD risk than those with lower individual incomes who lived in low-income neighborhoods (18). Individuals with high income who lived in neighborhoods with poor healthy food access had better cardiovascular profiles compared to individuals with low income living in high-income neighborhoods. This confirms that the perceived association between food deserts and CVD risk is partly due to individual income status rather than access to healthy foods. Further, another study suggested that there is a similar relationship between SES and CVD and found that mortality from CVD is higher in individuals with lower education levels and lower occupational class (19). The correlation between lower income and heightened risk of CVD, with CVD increasing the risk for serious illness related to infection from COVID-19, suggests an inverse correlation between income and COVID-19 health complications.

### Role of Food Deserts on Hypertension and Chronic Kidney Disease

Low income has also been associated with hypertension and CKD. Healthier foods, such as fruits and vegetables, tend to be costlier. This makes it hard for low-income families to afford healthy diets. Individuals have access to high amounts of processed meats and fats instead of fruits and vegetables in low-income neighborhoods and food deserts. A qualitative study done by Suarez et al. has revealed that 80.3% of participants living in food deserts and those with low incomes reported that they “always” or “most of the time” have fruit available at home (20). This is compared to 87.0% of participants that do not live in food deserts and are in the highest income category. 71.6% of participants living in food deserts and those with low incomes reported that they “always” or “most of the time” have dark green vegetables available at home compared to 82.0% that do not live in food deserts and are in the highest income category (20). Qualitatively, family income demonstrated a stronger association with diet, blood pressure, and CKD than living in a food desert (20).

The same study also found that serum carotenoids were low in individuals living in food deserts and individuals with low incomes (20). Carotenoids are a measure of fruit and vegetable intake. They also found that average protein, potassium, sodium, calcium, and magnesium intake were lower among individuals living in food deserts and individuals with low incomes. Measuring levels of these minerals gives insight into the measure of dietary acid load in an individual's body. Low levels of these minerals indicate a higher measure of dietary acid load (21). Foods rich in protein (meat, cheese, eggs, etc.) increase acid production in the body. Fruits and vegetables lead to base production. Diets high in acid induce metabolic acidosis, which can lead to hypertension, CKD, insulin resistance, diabetes, and other complications (20). A high dietary acid load has also been linked to obesity (22).

### Role of SDOH on Obesity

Food deserts contain more fast food restaurants than grocery stores. Individuals living in a food desert tend to have a poor diet, which increases the risk of obesity (23). Obesity is classified as a BMI greater than or equal to 40 by the CDC (8). Individuals living outside of food deserts have better access to grocery stores and are more likely to have diets consisting of more fruits and vegetables. These individuals are less likely to be at risk of obesity (23). Individuals who are obese are at higher risk of being diagnosed with a breathing disorder known as obesity hypoventilation syndrome, also known as Pickwickian syndrome. It is not clearly understood why this syndrome affects obese individuals, but it is thought that extra fat on the neck, chest, or abdomen may make breathing deeply difficult. This leads to a buildup of carbon dioxide and decreased amounts of oxygen in the blood. Hormones that affect breathing pattern may also be secreted in response to difficulty in breathing (24).

Body mass index (BMI) is calculated by dividing a person's weight in kilograms by the square of their height in meters (kg/m^2^). BMI is a screening tool used to determine whether a person is in a healthy weight range, overweight, or obese. A BMI of <18.5 classifies a person as underweight. BMI between 18.5 and <25 is normal. BMI between 25.0 and <30 puts an individual in the overweight range. BMI 30.0 or higher puts an individual in the obese range (25).

A study with 24 patients who tested positive with COVID-19 was conducted in Seattle. Of the 24 patients, 7 were classified as overweight and 13 as obese. The study showed that 85% of the obese patients required mechanical ventilation (26). Sixty-two percentage of the obese patients died from the virus. Sixty-four percentage of non-obese patients required mechanical ventilation, and 34% of them died from the virus (26). The percentages of requiring mechanical ventilation and deaths are clearly higher in obese individuals compared to non-obese individuals.

A BMI >40 was found to be the second strongest independent predictor of hospitalization in patients with COVID-19 at an academic hospital in New York City (27). A study in France that collected data from 124 patients who tested positive for COVID-19 reported that the ones who required mechanical ventilation were those who had a BMI greater than or equal to 35. The study mentions that the reason behind why patients usually require mechanical ventilation is because of impaired respiratory mechanics, increased airway resistance, and impaired gas exchange (28). In obese individuals, respiratory problems include low respiratory muscle strength, possible due to the extra fat on the neck, chest, or abdomen as mentioned earlier, and low lung volumes due to the extra fat making it difficult to take deep breaths (24, 28). The study also concluded that the disease severity of COVID-19 increased with increasing BMI (28).

## Social and Community Context

### Discrimination

Unfair or unjustified socially structured actions against a certain group or population contribute to discrimination. These actions tend to favor the affluent and powerful population at the detriment of the impoverished population. Discrimination occurs at both the individual and structural level in health care (17). Individual discrimination includes negative interactions between a patient and a health care provider due to race, gender, etc. Negative interactions may limit health care resources and well-being of the patient. Structural discrimination is seen in the form of residential segregation according to race or ethnic groups, unequal job opportunities due to gender, unequal access to quality education, inequalities in incarceration, etc. Forms of structural discrimination can trickle down to affect individuals and populations in terms of health care. Residential segregation plays a major role in the inequalities observed between African Americans and Caucasian populations. African Americans are more likely to live in high-poverty neighborhoods than other Americans. High-poverty neighborhoods consist of low quality and poor schools, limited access to healthcare and jobs, weak social networks, high rates of crime, pollution, and congestion (29). Because of congestion in impoverished neighborhoods, it can be difficult to follow social isolation recommendations. Keeping physical distance from others may not be an option for some families. Many individuals living in poverty are also in a predicament during times like this when people are asked to work from home, because minorities and African Americans are more likely to hold jobs in professions in which it is not feasible to work from home (30). Many Latinos and African Americans are facing the dilemma of having to pay rent and putting food on the table vs. staying home and keeping their families healthy during this COVID-19 outbreak, as they are the ones who work in warehouses, food industry, construction, janitorial services, etc., and these are jobs that cannot be done from home (30). Though race and ethnicity data are available for only 35% of those who have fallen victim to the virus, discrimination is clearly evident in the existing data (31). New York City, the hardest hit city in the U.S., has had more Latinos per capita fall victim to COVID-19 than any other ethnic groups (29). Latinos make up 29% of New York City's population. Approximately 34% of COVID-19 deaths in New York City are of Latinos. African Americans make up 22% of the city's population and 28% of COVID-19 deaths (32). Overall, African Americans are 2.4 times more likely to die from this virus compared to their counterparts of other races. Broken down by state, the statistics are alarming. African Americans make up ~13% of the U.S. population, and their population as a whole has endured 32% of COVID-19 deaths. On the other hand, Caucasians are disproportionately facing deaths based on which U.S. state they reside in. As a whole, Caucasians are less likely to die than expected at 0.8 times their counterparts (32).

### Community Involvement and Social Cohesion

Social support is an important component of an individual's well-being. Social cohesion, one of the terms used to describe social relationships, describes how strong relationships are and whether there is a sense of solidarity among members of a community (14). Social capital, an indicator of social cohesion, measures the extent of shared group resources within a community, perceived fairness, perceived helpfulness, group membership, and trust (14). Researchers found these aforementioned measures of social capital to be inversely correlated with mortality (33). Social capital decreases as income inequality increases. It is believed that social capital is the element that relates income inequality and mortality (14). Social cohesion is associated with lower neighborhood violence, better self-rated health, and less stress/anxiety. Stress has many impacts on the body, including on the immune, cardiovascular, and neuroendocrine systems. A study has showed that higher amounts of social support were associated with lower levels of atherosclerosis in women predisposed to a higher risk for CVD (34). Another study in California demonstrated that social support among Mexican adults served as a barrier against the detriments of the discrimination they faced (35).

It is evident that people and communities have come together during this difficult time. Medical students have been suspended from clinical clerkships, which prevents students from all patient care activities. Across the nation, medical students have been helping out resident physicians and attending physicians who are on the front-line with childcare, pet care, and running errands. Medical students from the University of Nebraska Medical Center have also been utilizing time off from clinical clerkships by volunteering in the community. Those who know how to sew have been sewing masks for front-line workers due to a shortage of personal protective equipment (PPE). Individuals have been running errands for the elderly who are more vulnerable to falling ill with the virus. During times of a global health crisis in which there is a call for social isolation, such as the one we face currently with the COVID-19 pandemic, it is important to find ways to maintain communication and social cohesion to preserve each other's well-being.

## Neighborhood and Built Environment

### Access to Healthy Foods

Food is an essential human need. It plays a major role in an individual's health and quality of life. Consumption of healthy foods is associated with lower risk of chronic health conditions. A healthy diet consists of a myriad of fruit, vegetables, grains, protein-rich foods (seafood, lean meats, poultry, legumes, soy products, eggs, etc.), and fat-free or low-fat dairy. Poor diet and nutrition have been linked to chronic conditions, such as CVD, hypertension, diabetes, and even cancer (36).

The individual components of the neighborhood and built environment domain of SDOH are intertwined and affect one another. There are many barriers to the access of healthy foods. Transportation, another component of the neighborhood and built environment domain, plays a major role in the access to healthy foods. A study from 2012 to 2013 found that on average, the nearest grocery store to households in the U.S. was 2.19 miles (36). This makes it difficult for those without their own vehicles or access to public transportation to make a trip to the grocery store.

Food deserts are neighborhoods that are defined as low income areas with little access to healthy foods by the U.S. Department of Agriculture (USDA) (23). These neighborhoods are more likely to contain fast food restaurants and convenience stores than grocery stores. Fast food restaurants and convenience stores contain options that are of lower quality and more unhealthy foods (higher saturated and *trans*-fat and higher calories). Individuals living in food deserts are more likely to have poor diets and nutrition as a result. Compared to Caucasian neighborhoods, African American and Latino neighborhoods are more likely to contain a higher amount of fast food restaurants and convenience stores. This explains why minority populations are more likely to have negative health outcomes than their racial counterparts. Living in a food desert puts an individual at a higher risk of obesity, which is discussed in another section.

Income also plays a role in access to healthy foods. Studies have shown that low-income families depend on cheap foods that happen to be low in nutrient density. Healthy foods, such as fresh fruits and vegetables, are usually more expensive than processed foods. Those who cannot afford fresh foods opt to the processed foods option, which is unhealthy (36). It is important to recognize food deserts and communities that do not have access to healthy foods, especially during a pandemic, when supplies may be in shortage to begin with. If supplies are in shortage, it will be difficult for those who have limited access to healthy foods or food in general to maintain their diet and nutrition altogether. Individuals will also have to make more trips to grocery stores to obtain groceries, which can put them at risk of acquiring the virus. Minority and low-income populations living in food deserts may face more difficulty accessing healthy foods during the COVID-19 pandemic due to customers overbuying and stocking groceries. This could be more of a problem in areas that are food deserts compared to affluent areas.

### Neighborhood/Environmental Conditions

Air quality, water quality, pollution, housing, and access to green space can all be discussed under this section. Health disparities due to neighborhood and environmental conditions can be understood by studying how certain population ends up in certain geographic locations. There is an association between racial minorities and geographic location of their residences. Latinos and African Americans are more likely to live in neighborhoods that have higher exposure to pollution from airborne particles such as chlorine, aluminum, and carbon (37). This is due to the fact that high-poverty neighborhoods in which Latinos and African Americans live are more likely to be located near factories, refineries, and landfills that emit pollutants. For a third of Americans, groundwater was found to be the major source of drinking water. Groundwater near factories, refineries, and landfills tends to be polluted with hazardous wastes (37).

Researchers have suggested that air pollution can make individuals more vulnerable to acquiring COVID-19. They reason that pollution particles are acting as vehicles for the virus, which makes it easier for the virus to be transmitted from person-to-person. Researchers say that air pollution may have worsened the outbreak. This may be due to the fact that air pollution weakens the immune system, which decreases one's ability to fight infections (37). A study recently found that an increase in the size of pollution particles, referred to as PM_2.5_, can have an effect on the spread of COVID-19. The study found that an increase of 1 microgram per cubic meter was associated with an 8% increase in deaths related to COVID-19 (38).

Safety also plays a major role in health. High-poverty neighborhoods are more likely to contain higher rates of crime, which decreases safety of community members. People are more likely to utilize available green space for walking, running, or exercising. Another issue in high-poverty neighborhoods is availability of green space. These neighborhoods are crowded to the point where there is minimal green space available for residents. Social distancing has been the key to flattening the curve and decreasing transmission of COVID-19. In neighborhoods that are crowded, social distancing may not be feasible. This puts individuals living in crowded neighborhoods at a higher risk of becoming ill with the virus, as well as increases the rate of transmission of the virus.

Low-income families tend to live in public housing of poor quality (39). A study found that public housing was found to have several infestations with cockroaches, mice, rats, etc. (40). Mold, lack of air conditioning, and tobacco smoke were also a common find (39). This study also found that 22% of children who lived in public housing were diagnosed with asthma compared to only 7% of those living in single-family homes (40). Low-income families may be at a higher risk of acquiring COVID-19.

## Education

### High School Graduation

For most jobs and higher educational degrees, a high school diploma is required (41). Without a high school education and diploma, job opportunities become slim. Lack of or less job opportunities can lead to poverty. Poverty can lead to negative health outcomes as discussed previously. The home and school environment is the major determinants of whether a student will graduate high school. Studies have found that students with parents who are not involved in their education are more likely to drop out of high school. Schools with higher crime rates are more likely to higher dropout rates (41).

Students from low-income households are more likely to attend low quality schools and have less access to educational resources. During the COVID-19 pandemic, schools have had to switch to online education. These children may not have access to computers, or internet. This means that children from high-income families are at an advantage when it comes to learning remotely, while children from low-income families are losing ground. Children with parents who are educated and have obtained higher educational degrees may encourage their children to keep pursuing their academic work (41). Non-educated parents may undervalue education compared to educated parents and downplay the importance of maintaining academic standards for their children. This does not make the educated parents better than the non-educated parents. Rather, it is a matter of being aware of and having experiences of how to navigate situations keeping in mind that education is important regardless of the hardships. Children with non-educated parents may not be getting the support that children with educated parents are getting while having to go to school online during this pandemic. Some children are stimulated to do well in a classroom setting and having to participate in distance learning may impact their academic merit.

### Language and Literacy

Individuals with lower levels of education and minorities are more likely to have limited English-speaking skills and lower literacy. Those with language and literacy barriers were noted to have worse health status, chronic health conditions, lack health insurance, and have difficulty following medication directions (42).

The U.S. is home to many who speak a language other than English. A new initiative, called the “COVID-19 Health Literacy Project,” started by medical students and physicians at Harvard Medical School, is intended to bridge the language barrier gap. This initiative has translated important COVID-19 information in over 35 languages (43). Languages that information can be translated into include Arabic, Bengali, Chinese, Dutch, Filipino, German, Greek, Gujarati, Japanese, Hindi, and many more. Information about the virus, prevention methods to avoid becoming ill with the virus, and treatment options available are included in the fact sheets. This has made it possible to educate the public even with existing language barriers. Creating awareness of the virus and educating the public about the situation and what precautions to take is an important step toward controlling the spread of the illness.

## Economic Stability

### Employment

The level of education one obtains is a major determinant of the type of job one has, the income they earn, and benefits such as health insurance, paid sick leave, and parental leave (44). Racial disparities also exist in the workplace. Caucasians are more likely to hold white-collar clerical jobs, while African Americans and minorities are more likely to hold blue-collar service jobs (44). Discrimination in the workplace can lead to stress, anxiety, depression, and negative health outcomes. Individuals who are unemployed are more likely to have stress-related conditions such as CVD, hypertension, and diabetes, which are all risk factors for COVID-19 (44).

The U.S. economic activity has slowed down with stay-at-home and quarantine orders. Many people have lost income by losing their job, having their salary reduced, or being put on unpaid leave (45). Approximately 33.5 million Americans have filed for unemployment aid in the last seven weeks (46). Approximately 61% of Hispanics and 44% of African Americans have reported that they have faced wage or job loss due to the COVID-19 pandemic compared to 38% of Caucasians (47). These percentages have increased from 49, 36, and 29%, respectively, since March (47). Unemployment or job loss means individuals do not have or lose their employer-sponsored health insurance. Congress has allowed uninsured individuals to be tested for COVID-19, however, treatment of the virus is not covered (48).

To address the economic downfall, the President of the United States signed the Coronavirus Aid, Relief, and Economic Security Act (CARES) stimulus bill into legislation on March 27, 2020 (45). The stimulus bill provides a payment of $1,200 for each U.S. citizen or U.S. resident alien with an income of $75,000 or less (49, 50). $500 is added to the $1,200 for each dependent child (45). Though it may seem simple, the criteria that have to be met to receive a stimulus check are numerous and complicated. A schedule for distribution of stimulus checks has not been established. As of now, one stimulus check has been sent out to qualifying individuals (50). The President and Congress have mentioned releasing a second check; however, nothing is set in stone (49–51). One check of $1,200 may not be enough for most families. This could certainly be a hindrance for families to eat healthy foods, as they will have to use the money wisely until either another check will be distributed, or the pandemic comes to an end and people can return to work.

There is a fine line between trying to decrease the spread of COVID-19 and preventing the progression of economic decline. It is evident that social distancing and quarantine methods are helping to flatten the curve, however, at the expense of the country's economic stability. Social distancing was recommended early on by each state's governors, and then a lockdown followed. Two states, Georgia and Idaho, demonstrate the rise in incidence of cases in the months of March and April, a decline toward the end of May, and rise again in the months of June and July (51, 52).

**Figure 2:**
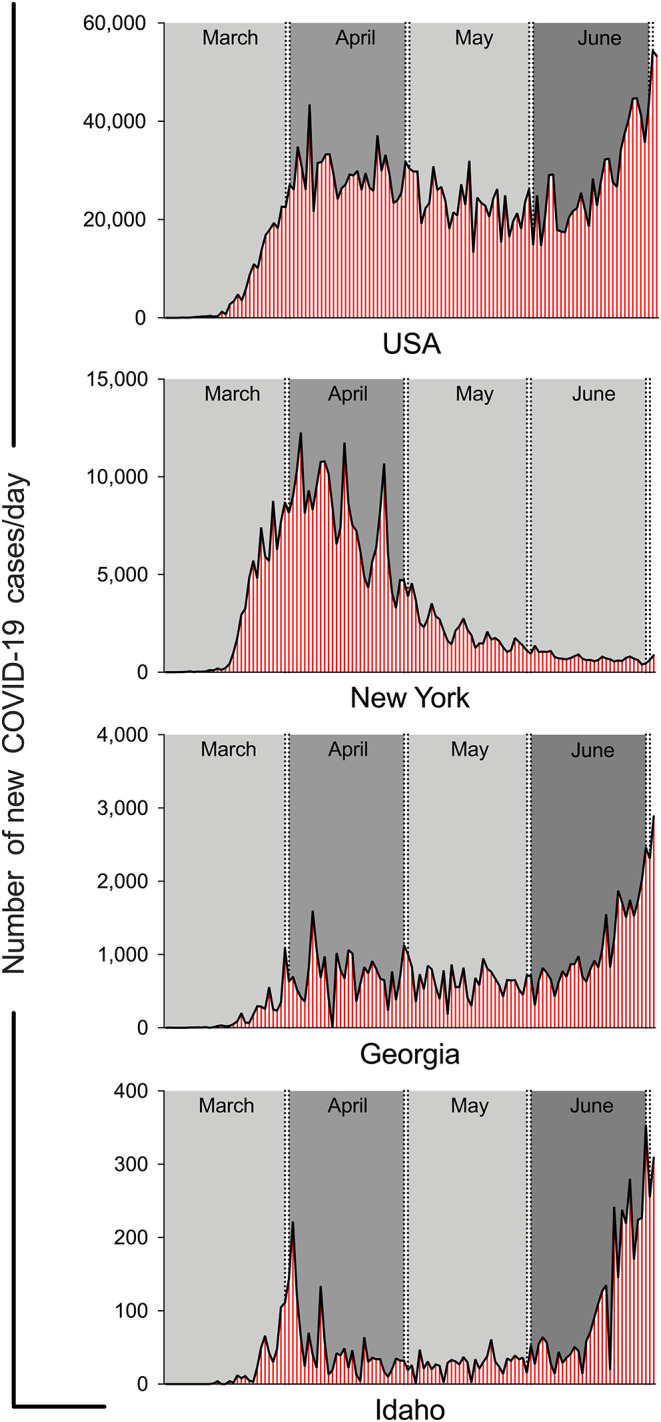
The wax and wane in new cases of COVID-19 per day in USA, New York, Georgia and Idaho. The graphs were generated using the online data form CDC and John Hopkins web sites.

Georgia's governor issued a lockdown on April 3, 2020, and Idaho's governor issued a lockdown on March 25, 2020 (53, 54). During lockdown, non-essential workers were directed to stay at home and only go out to the grocery store or to a pharmacy if needed. Social distancing was to be followed strictly during lockdown. Georgia's lockdown was lifted on April 24, 2020 (55). At the end of April, Georgia saw a slight increase in incidence of cases. By mid-June, the incidence is higher in Georgia than before lockdown was implemented, and it is only increasing. Idaho's governor, on the other hand, issued a lockdown on March 25, 2020 (54). There was a rise in incidence at the beginning of April and then a decline by mid-April. Idaho's lockdown was lifted on April 30, 2020 (56). The incidence was <40 cases in Idaho from mid-April to the beginning of June. Since June 1, 2020, the incidence is on the rise, and it is higher in June and July compared to when lockdown was implemented in March. The incidence of COVID-19 cases overall in the U.S. is shown in Figure 2 (57). It is evident that incidence is once again on the rise as lockdowns have been lifted across the nation and social distancing is no longer being followed as strictly as during the lockdowns (Figure 2). It is understandable that the nation's economy is an important consideration when implementing a lockdown across the nation. We will have to wait and see what the future holds for our nation's economy while we try to eradicate COVID-19.

## Conclusions

Pandemics are more of a social problem than a healthcare problem. The population that lives in poverty and in neighborhoods that are overcrowded with poor maintenance and sanitation is being disproportionately affected by COVID-19. It is imperative to provide additional aid for low-income families, such as the stimulus check. This is especially important during times of disease outbreaks, as this is a vulnerable population that is at risk for serious illness. The root cause of being a part of the vulnerable population at risk during outbreaks comes down to income level and racial/ethnic identification. Lower income has been associated with poor dietary intake and habits. Minority groups, such as Latinos, and African Americans are at a disadvantage due to individual and structural discrimination, and they are more likely than their Caucasian counterpart to be vulnerable to negative health outcomes. Therefore, it is evident that the SDOH have been overlooked during this pandemic. Dr. Richard Clarke Cabot, an American physician, was the first in the U.S. to consider socioeconomic, family, and psychological factors when practicing medicine (https://www.ncbi.nlm.nih.gov/books/NBK702/). He observed that there was a correlation between lower socioeconomic status of patients and their probability of succumbing to illness. Historical reports have shown that poverty, inequalities, and SDOH facilitate the spread of infectious diseases. Inequalities in health and healthcare can further add to disparities in morbidity and mortality. Quinn et al. suggested that existing studies of influenza pandemics have not recognized the importance of health inequalities nor have they attempted to analyze differences in socioeconomic factors and how they impact health during times of a health emergency (58). Therefore, it is imperative to respond rapidly and effectively during times of a health emergency. In order to achieve that, it is crucial to be educated about all of the factors that may play a role in health and healthcare before an outbreak of disease even occurs. Having insight into factors that play a role in health and healthcare, such as SDOH, can facilitate access to medical and non-medical resources to those who are socioeconomically disadvantaged. Public education and creating awareness of the severity of the virus is also important. Awareness of the disadvantaged population that is more vulnerable than the average individual and the rapid spread of COVID-19 should motivate individuals to reduce exposure to others to stop the spread of the disease. The key to fighting an outbreak is to take into account the various factors that play a role in the well-being of a nation. Appropriate and timely education, health care, and social services can be effective measures taken to address outbreaks, such as COVID-19.

Integrating SDOH into efforts to eliminate disparities in health and healthcare can be the solution to reducing disease globally. This can be done through the assembly of an interdisciplinary team that consists of health care professionals, public health professionals, anthropologists, sociologists, researchers, governments, National Institute of Health (NIH), Center for Diseases Control (CDC), World Health Organization (WHO), and others, who can all contribute to analyzing and understanding the various factors that play a role in causing health disparities in populations that already face socioeconomic inequalities. It is also crucial to assess what actions and measures were taken correctly and what went wrong during this pandemic, so that, we will be prepared to handle things in a more efficient manner if any future pandemics arise.

Every person, regardless of where they live, what race they are, and what income they have, should have equal opportunities to stay healthy. By incorporating SDOH into preventing the spread of disease and to approach patient care in a holistic manner, the unfair differences can be minimized socially and economically.

## Author Contributions

SS designed and drafted/wrote the manuscript. AA referencing and edited the manuscript. KC edited the manuscript. SB designed and edited/wrote the manuscript. All authors contributed to the article and approved the submitted version.

## Conflict of Interest

The authors declare that the research was conducted in the absence of any commercial or financial relationships that could be construed as a potential conflict of interest.
